# Induction of microRNA-214-5p in human and rodent liver fibrosis

**DOI:** 10.1186/1755-1536-5-12

**Published:** 2012-08-01

**Authors:** Masashi Iizuka, Tomohiro Ogawa, Masaru Enomoto, Hiroyuki Motoyama, Katsutoshi Yoshizato, Kazuo Ikeda, Norifumi Kawada

**Affiliations:** 1 Department of Hepatology, Graduate School of Medicine, Osaka City University, 1-4-3, Asahimachi, Abeno, Osaka 545-8585, Japan; 2 Center for the Advancement of Higher Education, Faculty of Engineering, Kinki University, 1, Takaya Umenobe, Higashi-Hiroshima City, Hiroshima 739-2116, Japan; 3 PhoenixBio Co. Ltd., Hiroshima, Japan, 3-4-1, Kagamiyama, Higashi-Hiroshima City, Hiroshima 739-0046, Japan; 4 Department of Anatomy and Regenerative Biology, Graduate School of Medicine, Osaka City University, 1-4-3, Asahimachi, Abeno, Osaka 545-8585, Japan

**Keywords:** Collagen, Hepatocyte, Non-coding RNA, Stellate cell, Transforming growth factor-β

## Abstract

**Background:**

miRNAs are non-coding RNAs that regulate gene expression in a wide range of biological contexts, including a variety of diseases. The present study clarified the role of miR-214-5p in hepatic fibrogenesis using human clinical tissue samples, livers from rodent models, and cultured hepatic stellate cells.

**Methods:**

The expression of miR-214-5p and genes that are involved in liver fibrosis were analyzed in hepatitis C virus-infected human livers, rodent fibrotic livers, a human stellate cell line (LX-2), and the cells from intact mouse livers using real-time PCR. The effect of miR-214-5p overexpression in LX-2 cells on cell function was investigated. Twist-1 expression in the liver tissues of mouse models and primary-cultured stellate cells was also analyzed.

**Results:**

miR-214-5p was upregulated in human and mouse livers in a fibrosis progression–dependent manner. miR-214-5p expression increased during the culture-dependent activation of mouse primary stellate cells and was significantly higher in stellate cells than in hepatocytes. The overexpression of miR-214-5p in LX-2 cells increased the expression of fibrosis-related genes, such as matrix metalloproteinase (MMP)-2, MMP-9, α-smooth muscle actin, and transforming growth factor (TGF)-β1. TGF-β stimulation induced miR-214-5p in LX-2 cells. Twist-1 was increased in fibrotic mouse livers and induced during mouse stellate cell activation.

**Conclusion:**

miR-214-5p may play crucial roles in the activation of stellate cells and the progression of liver fibrosis. Twist-1 may regulate miR-214-5p expression in the liver, particularly in stellate cells.

## Background

Liver fibrosis is a consequence of chronic liver trauma caused by hepatitis B or hepatitis C virus (HCV) infection, alcohol abuse, or steatohepatitis, which ultimately leads to liver cirrhosis, liver failure, and hepatocellular carcinoma [1]. Liver fibrosis is characterized by an abnormal accumulation of extracellular matrix (ECM) components, including types I and III collagen, laminin, and proteoglycans, in the liver parenchyma [2,3]. Transforming growth factor (TGF)-β, which is produced and released by activated macrophages and platelets at the site of local inflammation, is considered to play a primary role in the fibrotic process [3]. Hepatic stellate cells - which are localized in Disse’s space, store vitamin A and act as tissue-specific pericytes under physiological conditions - undergo activation and transformation into myofibroblast-like cells that express α-smooth muscle actin (α-SMA) during persistent inflammation. The activated stellate cells become an additional source of TGF-β and a principal producer of ECM components. However, the detailed molecular mechanisms of TGF-β production in these cells have not been determined [4].

miRNAs are 20 to 24 nucleotide non-coding RNAs that are involved in the post-transcriptional regulation of gene expression. Mature miRNAs are incorporated into an RNA-induced silencing complex that recognizes target mRNAs through imperfect base pairing with the miRNA. This action triggers the translational inhibition or destabilization of the target mRNA, which results in the regulation of crucial biological processes, such as development, differentiation, apoptosis and cellular proliferation [5,6]. Aberrant expression of miRNAs in tissues correlates with a variety of diseases, including proliferative vascular disease [7], cardiac disorders [8,9], polycystic kidney disease [10], and cancer [11,12]. Several miRNAs can be used as biomarkers for cancer [13,14] because miRNA expression patterns in human cancer are tissue specific [15].

miR-122 is the most abundant miRNA in the liver, where it regulates fat metabolism and the replication of HCV in hepatocytes and contributes to carcinogenesis [16,17]. miR-122 has been used as a novel biomarker for liver damage in rat models of hepatocellular injury caused by a methionine- and choline-deficient diet (MCDD), CCl_4_ or acetaminophen and bile duct ligation [18]. We previously reported that miR-29b regulates collagen expression by binding to the 3′-UTR of the type 1 collagen alpha 1 chain (Col1a1) and SP1 mRNAs [19], and miR-29b directly inhibits the activation of mouse stellate cells in primary culture [20]. It was recently reported that miR-19b suppresses the activation of stellate cells via the inhibition of TGF-β signaling by interacting with the type II TGF-β receptor [21].

miR-214-5p is a product of the 110 bp *miR-214* gene in the intron of the *Dynamin-3* gene on human Chromosome 1-NC_000001.10, which produces a mature miRNA with a sequence of ugccugucuacacuugcugugc [22]. TGF-β induces miR-214 expression in rat tubular epithelial cells and mesangial cells [23], and miR-214 interacts with Quaking to inhibit angiogenesis [24]. However, the pathophysiological roles of miR-214 remain largely unknown. Here, we report the upregulation of miR-214-5p in a fibrosis progression–dependent manner in HCV-infected human livers and in the livers of a rodent fibrosis model. The role of miR-214-5p in hepatic stellate cell activation is also discussed.

## Results

### miR-214 expression in chronic hepatitis C patients

**Figure 1:**
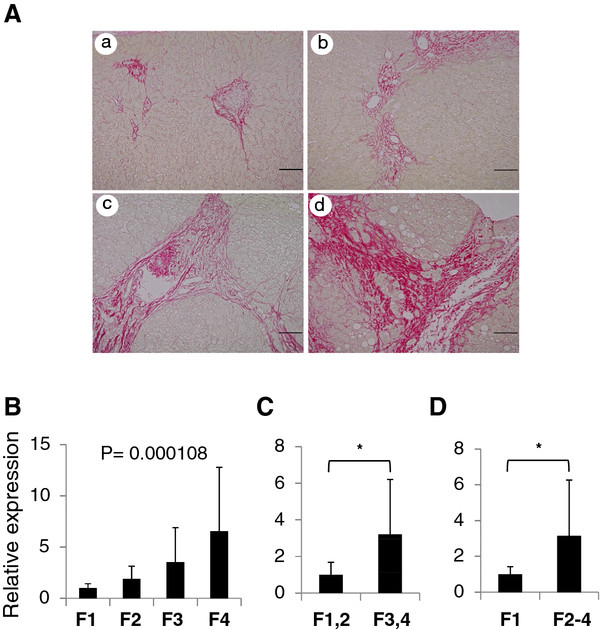
**miR-214-5p expression in the livers of patients with chronic hepatitis C virus infection.** miR-214-5p expression in the livers of 35 hepatitis C virus (HCV)-infected patients was analyzed using real-time PCR. **(A)** Representative sirius red staining of liver tissues with fibrosis stages F1-F4 of chronic hepatitis C patients included in this study. **(B)** miR-214-5p expression in samples from each stage of liver fibrosis (METAVIR scoring system). The numbers of patients in each stage were: F1, 17; F2, 8; F3, 8; and F4, 2. The Jonckheere-Terpstra test identified trends among classes. **(C)** Comparison of miR-214-5p expression in F1/F2 and F3/F4 samples. **(D)** Comparison of miR-214-5p expression between *F1 and F2-4. The levels of miR-214-5p expression in B, C and D are indicated relative to F1,* F1/F2 and F1, respectively. *P < 0.05.

We previously found that, using microRNA array analysis, miR-221/222 expression was upregulated in a fibrosis progression–dependent manner in human livers that are chronically infected with HCV [25]. In addition, we quantitatively confirmed the miR-214-5p expression levels in 35 HCV patients with individual stages of liver fibrosis (Figure 1A) using real-time PCR. We found that miR-214-5p expression increased according to the stage of fibrosis (*P* = 0.000108) (Figure 1B) and was significantly higher in patients with advanced liver fibrosis than in those with mild fibrosis (F1/F2 versus F3/F4: 3.2-fold, *P* < 0.05; F1 versus F2-4: 3.1-fold, *P* < 0.05) (Figure 1C,D). 

### miR-214 expression in a mouse model of liver fibrosis

**Figure 2:**
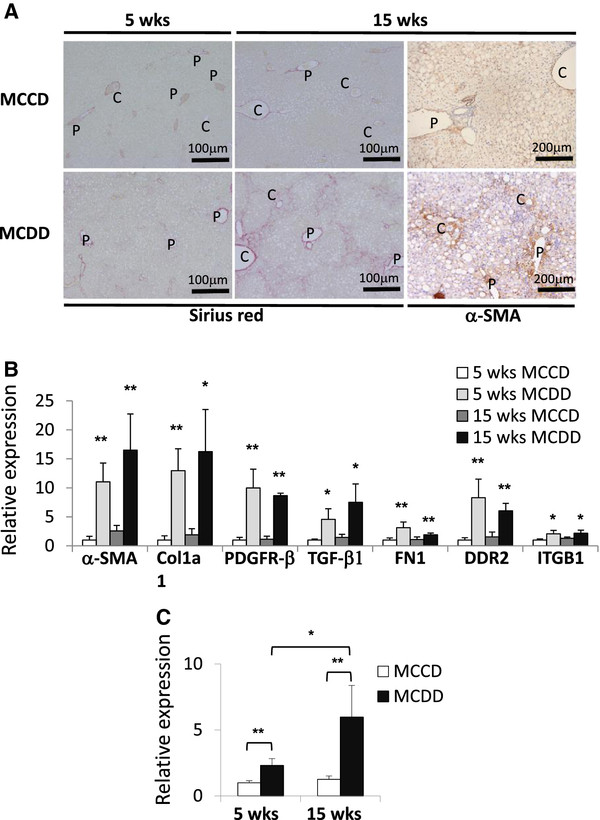
**miR-214-5p expression in mouse livers with fibrosis induced by an methionine- and choline-deficient diet.(A)** Sirius red staining (left and middle panels) and α-smooth muscle actin (SMA) immunostaining (right panels) of mouse liver tissues. Collagen deposition and an increase in α-SMA-positive cells were evident around the central vein area of the liver of mice that received the MCDD for 15 weeks. Scale bars, 100 μm (left and middle panels) and 200 μm (right panels). P, portal vein. C, central vein. **(B)** The mRNA expression of α-smooth muscle actin (α-SMA), the type 1 collagen alpha 1 chain (Col1a1), platelet-derived growth factor receptor (PDGFR)-β, transforming growth factor (TGF)-β1, fibronectin (FN)1, discoidin domain receptor (DDR)2, and β1 integrin (ITGB1) in fibrotic mouse livers was analyzed using real-time PCR. The results are expressed relative to mRNA expression at 5 weeks of the methionine- and choline-control diet (MCCD). *P < 0.05. **P < 0.01. **(C)** miR-214-5p expression in fibrotic mouse livers was analyzed using real-time PCR. The results are expressed relative to the expression of miR-214-5p at 5 weeks of the MCCD. *P < 0.05. **P < 0.01.

Liver fibrosis was induced by feeding mice a MCDD for 5 or 15 weeks and then compared with mice fed a methionine- and choline-control diet (MCCD). Sirius red staining and α-SMA immunostaining confirmed the time-dependent induction of fibrosis in the liver of MCDD-fed mice, especially around the central vein area (Figure 2A). The mRNAs of liver fibrosis factors, such as α-SMA, Col1a1, platelet-derived growth factor receptor (PDGFR)-β, TGF-β1, fibronectin (FN) 1, discoidin domain receptor (DDR) 2, and β1 integrin (ITGB1), were upregulated in the livers of MCDD-fed mice compared to MCCD-fed mice B(Figure 2). miR-214-5p expression was significantly higher in the livers of MCDD-fed mice than in control mice (2.1-fold, *P* < 0.01 at 5 weeks; and 4.8-fold, *P* < 0.01 at 15 weeks) (Figure 2C).

### miR-214 expression in a rat resolution model of liver fibrosis

**Figure 3:**
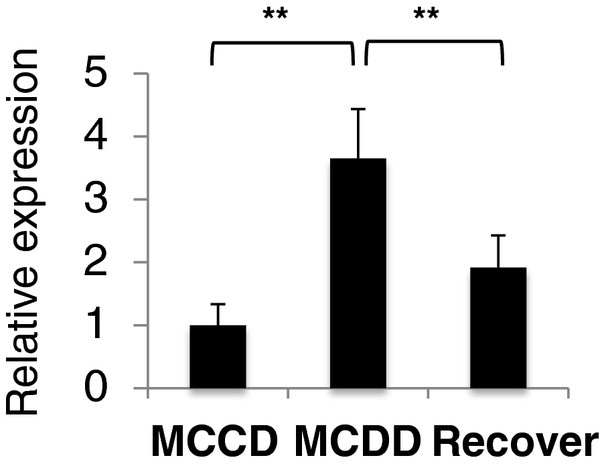
**miR-214-5p expression in rat livers with fibrosis induced by a methionine- and choline-deficient diet.** Expression of miR-214-5p in methionine- and choline-deficient diet (MCDD)-induced liver fibrosis and recovery phase in rats. The results are expressed relative to the expression of miR-214-5p in the livers of rats fed the methionine- and choline-control diet (MCCD) for 10 weeks. MCCD; rats fed MCCD for 10 weeks. MCDD; rats fed MCDD for 10 weeks. Recover; rats fed MCDD for 8 weeks followed by MCCD for 2 weeks. **P < 0.01.

We previously demonstrated the resolution of liver fibrosis with steatohepatitis in a rat model induced by giving MCDD; that is, rats received either MCCD for 10 weeks, MCDD for 10 weeks, or MCDD for 8 weeks followed by MCCD for the last 2 weeks (the last of the these being the recovery group) [26]. miR-214-5p expression was significantly greater in the livers of rats that received MCDD for 10 weeks than in those that received MCCD for 10 weeks. However, these levels returned to control levels in the livers of rats that received the MCDD diet for 8 weeks followed by the MCCD diet for 2 weeks, consistent with recovery from the fibrosis (Figure 3). These results clearly suggest a close correlation between miR-214-5p expression in the liver, fibrosis development, and fibrosis-related mRNA expression. 

### miR-214-5p expression in hepatic stellate cells

**Figure 4:**
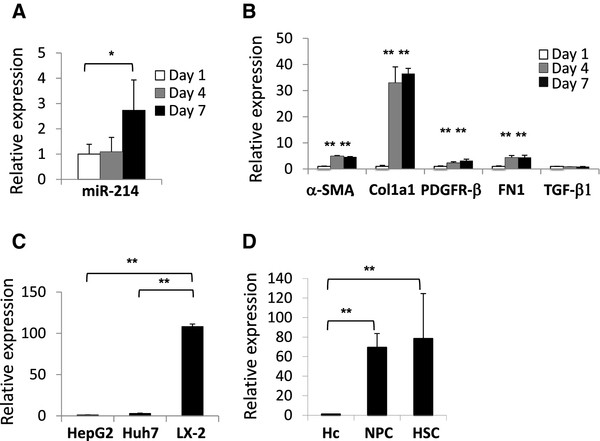
**miR-214-5p expression in liver cells, including stellate cells. (A)** miR-214-5p expression in mouse stellate cells during primary culture was analyzed using real-time PCR. Stellate cells were isolated from mouse livers and cultured for 1, 4 or 7 days. The results are expressed relative to the expression of miR-214-5p at day 1. *P < 0.05. **(B)** The expression of α- smooth muscle actin (α-SMA), the type 1 collagen alpha 1 chain (Col1a1), platelet-derived growth factor receptor (PDGFR)-β, fibronectin (FN)1 and transforming growth factor (TGF)-β1 mRNA in primary-cultured mouse stellate cells was analyzed using real-time PCR. The results are expressed relative to the expression of the same mRNA at day 1. **P < 0.01. **(C)** miR-214-5p expression in HepG2, Huh7 and LX-2 cells was analyzed using real-time PCR. The results are expressed relative to the expression of miR-214-5p in HepG2. **P < 0.01. **(D)** miR-214-5p expression in hepatocytes (Hc), the non-parenchymal cell (NPC) fraction, and the hepatic stellate cell (HSC) fraction. The cells were isolated from intact mouse livers. The miR-214-5p expression in Hc, the NPC fraction, and the HSC fraction was analyzed using real-time PCR. The results are expressed relative to the expression of miR-214-5p in hepatocytes. **P < 0.01.

We assessed the contribution of activated hepatic stellate cells to the increase in miR-214-5p in fibrotic mouse livers. miR-214-5p expression increased during the culture-dependent activation process in mouse stellate cells (2.7-fold increase at day 7 compared to day 1, *P* < 0.05) (Figure 4A). As expected, the induction of miR-214-5p was accompanied by an increase in the expression of α-SMA, Col1a1, PDGFR-β, and FN1 mRNA (Figure 4B). In addition, miR-214-5p expression was markedly higher in LX-2, a widely used human hepatic stellate cell line, than in human liver cancer cell lines such as HepG2 and Huh7 (108- and 39-fold, respectively) (Figure 4C).

We next isolated individual hepatocytes, non-parenchymal cells, and hepatic stellate cells from intact mouse livers to verify the cellular source of miR-214-5p. miR-214-5p was localized to non-parenchymal cells and hepatic stellate cells but expressed at negligible levels in hepatocytes (Figure 4D). These results suggest that miR-214 induction in fibrotic livers reflects the number and activation status of hepatic stellate cells.

### The effect of miR-214 overexpression on gene expression in stellate cells

**Figure 5:**
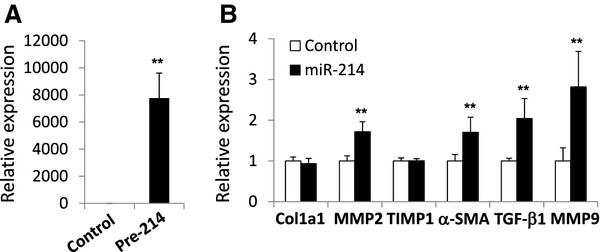
**Effect of miR-214 overexpression on mRNA expression in LX-2 cells. (A)** LX-2 cells were transfected with a miR-214 precursor or a negative control (control) at a final concentration of 50 nM and incubated for 24 hours. miR-214 expression was quantitated using real-time PCR. **(B)** The expression of fibrosis-related genes in LX-2 cells transfected with miR-214 precursors was analyzed using real-time PCR. The results are expressed as the expression relative to that in cells transfected with the control. **P < 0.01.

We investigated the effect of miR-214-5p overexpression on fibrosis-related gene expression in stellate cells to clarify the role of this miRNA in stellate cell activation. miR-214-5p was overexpressed in LX-2 cells by transfection with an miR-214 precursor. The overexpression of miR-214 significantly increased the expression of matrix metalloproteinase-2 (MMP-2), MMP-9, α-SMA, and TGF-β1 compared to cells transfected with control microRNA (1.7-, 2.8-, 1.7- and 2.0-fold, respectively; *P* < 0.01) (Figure 5). These results indicate the strong participation of miR-214 in the activation of stellate cells.

### Induction of miR-214 expression by TGF-β1

**Figure 6:**
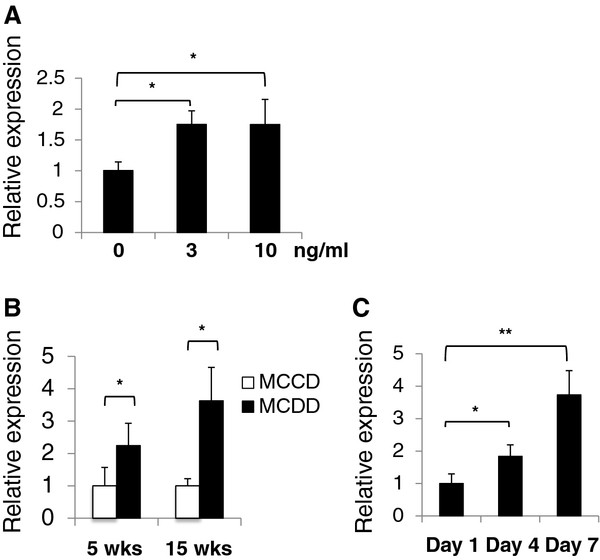
**Regulation of miR-214-5p expression. (A)** The effect of transforming growth factor (TGF)-β1 on miR-214-5p expression. LX-2 cells were treated with recombinant human TGF-β1 (3 or 10 ng/ml) for 24 hours in DMEM containing 0.1% fetal bovine serum (FBS). The results are expressed relative to miR-214 expression in cells that did not receive TGF-β1 treatment. *P < 0.05. **(B)** Twist-1 expression in the fibrotic livers of mice fed a methionine- and choline-deficient diet (MCDD). Twist-1 expression was analyzed using real-time PCR. The results are expressed relative to Twist-1 expression in methionine- and choline-control diet (MCCD) mice. *P < 0.05. **(C)** Twist-1 expression in primary-cultured mouse stellate cells. Twist-1 expression was analyzed using real-time PCR. The results are expressed relative to Twist-1 expression in cells on day 1. *P < 0.05, **P < 0.01.

TGF-β_1_ induces miR-214 expression in rat tubular epithelial cells and mesangial cells [23]. TGF-β_1_ is essential for hepatic stellate cell activation. We assessed the stimulatory effect of TGF-β on miR-214-5p expression in LX-2 cells. TGF-β_1_ (3 and 10 ng/ml) significantly stimulated miR-214-5p expression in LX-2 cells after 24 hours (1.75-fold, *P* < 0.05)(Figure 6A). In contrast, the expression of the miR-214/199a cluster is controlled by the transcription factor Twist-1 [22]. Real-time PCR analysis revealed that Twist-1 expression increased in the livers of mice that received MCDD compared to those of MCCD-fed mice (2.2-fold at 5 weeks, *P* < 0.05; and 3.6-fold at 15 weeks, *P* < 0.05) (Figure 6B). Twist-1 mRNA expression was also induced in a time-dependent manner after culture initiation in primary-cultured mouse stellate cells (Figure 6C). 

## Discussion

This is the first report to show that miR-214-5p is involved in organ fibrogenesis, specifically in the liver. miR-214 has previously been predicted to be a key molecule in proliferation in breast [27] and ovarian cancer cells [28], tumor progression in melanoma [29], and growth in HeLa cells [30]. miR-214 and miR-199a are encoded in a region that contains an E-box DNA promoter sequence [22]. A transcription factor, Twist-1, binds to the E-box region, regulating miR-214 and miR-199a expression [22]. The present study showed that miR-214 expression is upregulated in a fibrosis progression-dependent manner in the livers of patients with chronic HCV infection and in mice with diet-induced steatohepatitis (Figures 1 and 2). We previously reported an increase in miR-199a in the fibrotic livers of patients with chronic HCV infection [25], and similar findings have been reported by others [31-33]. These data and the upregulation of Twist-1 in MCDD-induced mouse liver fibrosis (Figure 4) suggest that Twist-1 controls the expression of the miR-214/199a cluster in the liver. Further studies will be needed to clarify the possible involvement of Twist-1 in the expression of miR-214-5p in LX-2 cells.

The present study revealed that miR-214-5p overexpression in LX-2 cells significantly increased MMP-2, MMP-9, α-SMA, and TGF-β1 mRNA expression. The overexpression of miR-199a in LX-2 cells triggers the upregulation of tissue inhibitor of metalloproteinase (TIMP)-1, Col1a1, and MMP-13 mRNA [34]. These results suggest that the miR-214/199a cluster plays a primary role in stellate cell activation. However, an understanding of the precise molecular events involved requires further research.

Conversely, the overexpression of miR-214-5p in LX-2 cells did not alter the expression of MAPK/Erk kinase 3 (MEK3), transcription factor AP-2 gamma (TFAP2C) [29], Plenxin-B1 [30], c-Jun N-terminal kinase 1 (Jnk1) [34], phosphatase and tensin homolog (PTEN) [35], enhancer of zeste homolog 2 (Ezh2) [36], and Quaking mRNA [24], which had been reported to be targets of miR-214 (MEK3: 0.72- to 0.77-fold, Jnk1: 1.05- to 1.20-fold, PTEN: 0.97- to 1.12-fold, Plenxin-B1: 0.99-fold, Ezh2: 0.96-fold, TFAP2C: 0.94-fold, and Quaking: 0.88- to 1.18-fold change compared with cells transfected with control miRNA). The PTEN 3′-UTR did not interact with miR-214-5p in a luciferase reporter assay in LX-2 cells (data not shown). We also found that miR-214-5p overexpression had a negligible effect on LX-2 proliferation and migration. Therefore, the mRNA targets of miR-214-5p in LX-2 cells are not identical to those in previous reports.

## Conclusions

We report an increase in miR-214-5p in liver fibrosis in humans and mice and the possible association of miR-214-5p with stellate cell activation. miR-214 expression in stellate cells is regulated by TGF-β and possibly by the transcription factor Twist-1. These results should be pursued further to identify the role of miR-214-5p in liver fibrogenesis and to develop a biomarker that reflects the stage of liver fibrosis more accurately than a pathological staging score.

## Methods

### Ethics Statement

The Ethics Committee of the Osaka City University Graduate School of Medicine approved this study (Approval No. 1358), which complied with the principles of the Declaration of Helsinki (2008 revision). All of the patients provided written, informed consent.

### Liver biopsy specimens

Liver biopsy specimens were obtained from 35 patients with chronic HCV (genotype 1) infection as described previously [25]. The stage of liver fibrosis was evaluated using the METAVIR scoring system [37]. Normal liver tissues were taken as control samples from four patients who underwent resection for metastatic liver tumors.

### Animals

Eight- to 12-week-old male C57BL/6N mice were purchased from Japan SLC, Inc. (Shizuoka, Japan). All animal procedures were performed according to the guidelines of the Osaka City University and Faculty of Medicine Animal Research Committee and were approved by the committee. The mice received either a MCDD (n = 7, MP Biomedicals, Solon, OH, USA) or a MCCD (n = 7, MP Biomedicals) for 5 or 15 weeks, as previously described [26]. A similar protocol was followed in rats purchased from Japan SLC, Inc. Rats received MCCD for 10 weeks, MCDD for 10 weeks, or MCDD for 8 weeks followed by MCCD for the last 2 weeks (the last of the these being the recovery group) [26].

### Cells

LX-2 cells (donated by Dr Scott Friedman [38]) and Huh7 cells were maintained in plastic culture plates in DMEM (Sigma Chemical Co., St Louis, MO, USA) supplemented with 10% fetal bovine serum (FBS) (Invitrogen, Carlsbad, CA, USA). HepG2 cells (JCRB1054) were obtained from the Health Science Research Resources Bank (Osaka, Japan) and maintained in plastic culture plates in Minimum Essential Medium (Invitrogen) supplemented with 10% FBS, 1 mM sodium pyruvate (Invitrogen), and 1% non-essential amino acids (Invitrogen). Primary hepatic stellate cells and hepatocyte-rich and Kupffer cell-rich fractions were prepared from mouse livers according to the previously reported method [39].

### Histochemistry and immunohistochemistry

The sections were stained with 0.1% (w/v) Sirius red in a saturated aqueous solution of picric acid (Direct Red 80; Aldrich, Milwaukee, WI, USA) for 1 hour at room temperature to visualize collagen fibers. Immunostaining for α-SMA was performed as previously described [25]. Mouse liver tissue was fixed in 10% formaldehyde, embedded in paraffin, and cut into 4 μm thick sections.

### Quantitative real-time PCR

Gene expression was measured by real-time PCR using cDNA, real-time PCR Master Mix Reagents (Toyobo, Osaka, Japan), and gene-specific oligonucleotide primers (Table 1) in an ABI Prism 7500 Real-Time PCR System (Applied Biosystems, Foster City, CA, USA), as previously described [25]. 

**Table 1:** List of primers

**Gene name**		**Sequence from 5′ to 3′**
Mouse GAPDH	F	TGCACCACCAACTGCTTAG
	R	GGATGCAGGGATGATGTTC
Mouse α-SMA	F	TCCCTGGAGAAGAGCTACGAACT
	R	AAGCGTTCGTTTCCAATGGT
Mouse Col1a1	F	CCTGGCAAAGACGGACTCAAC
	R	GCTGAAGTCATAACCGCCACTG
Mouse PDGFR-β	F	GCGTATCTATATCTTTGTGCCAGA
	R	ACAGGTCCTCGGAGTCCAT
Mouse TGF-β1	F	GCAACATGTGGAACTCTACCAGAA
	R	GACGTCAAAAGACAGCCACTC
Mouse FN1	F	GATGCCGATCAGAAGTTTGG
	R	GGTTGTGCAGATCTCCTCGT
Mouse DDR2	F	CGAAAGCTTCCAGAGTTTGC
	R	GCTTCACAACACCACTGCAC
Mouse ITGB1	F	CAACCACAACAGCTGCTTCTAA
	R	TCAGCCCTCTTGAATTTTAATGT
Mouse Twist-1	F	AGCTACGCCTTCTCCGTCT
	R	TCCTTCTCTGGAAACAATGACA
Human GAPDH	F	GCACCGTCAAGGCTGAGAAC
	R	TGGTGAAGACGCCAGTGGA
Human Col1a1	F	CCCGGGTTTCAGAGACAACTTC
	R	TCCACATGCTTTATTCCAGCAATC
Human MMP2	F	TGACATCAAGGGCATTCAGGAG
	R	TCTGAGCGATGCCATCAAATACA
Human TIMP1	F	GGATACTTCCACAGGTCCCACAA
	R	CTGCAGGTAGTGATGTGCAAGAGTC
Human α-SMA	F	GACAATGGCTCTGGGCTCTGTAA
	R	CTGTGCTTCGTCACCCACGTA
Human TGF-β1	F	AGCGACTCGCCAGAGTGGTTA
	R	GCAGTGTGTTATCCCTGCTGTCA
Human MMP9	F	TCGAACTTTGACAGCGACAAGAA
	R	TCAGTGAAGCGGTACATAGGGTACA

α-SMA, α-smooth muscle actin; Col1a1, the type 1 collagen alpha 1 chain; DDR, discoidin domain receptor; F, forward primer; FN, fibronectin; GAPDH, glyceraldehyde-3-phosphate dehydrogenase; ITGB1, β1 integrin; MMP, matrix metalloproteinase; PDGFR, platelet-derived growth factor receptor; R, reverse primer; TIMP, tissue inhibitor of metalloproteinase; TGF, transforming growth factor.

### Transforming growth factor-β1 stimulation of LX-2 cells

LX-2 cells were seeded on 24-well plates in DMEM supplemented with 10% FBS at a density of 2 × 10^5^ cells/ml. The cells were cultured for 14 hours, and the medium was changed to DMEM supplemented with 0.1% FBS plus TGF-β1 (3 or 10 ng/ml). The culture was continued for an additional 24 hours.

### Transient transfection of miRNA precursors

miR-214-5p precursors and negative control miRNA were transfected into LX-2 cells using Lipofectamine 2000 (Invitrogen) at a final concentration of 50 nM, as described previously [20,25]. The cells were collected after 24 hours, and total RNA was extracted.

### Statistical analysis

The data shown in the bar graphs represent the means ± SD of at least three independent experiments. Statistical analysis was performed using the Student’s *t*-test. The Jonckheere-Terpstra test was used to compare differences between the four groups in the progressive stages of liver fibrosis. *P* < 0.05 was considered statistically significant.

## Abbreviations

bp: base pair; Col1a1: the type 1 collagen alpha 1 chain; DDR: discoidin domain receptor; DMEM: Dulbecco’s modified Eagle’s medium; ECM: extracellular matrix; FBS: fetal bovine serum; FN: fibronectin; HCV: hepatitis C virus; ITGB1: β1 integrin; MCCD: methionine- and choline-control diet; MCDD: methionine- and choline-deficient diet; miRNA: microRNA; MMP: matrix metalloproteinase; PCR: polymerase chain reaction; PDGFR: platelet-derived growth factor receptor; α-SMA: α-smooth muscle actin; TGF: transforming growth factor; UTR: untranslated region.

## Competing interests

KY is an employee of PhoenixBio Co. Ltd as an academic advisor. There is no direct financial benefit to KY for the publication of this manuscript. All other authors declare that they have no competing interests.

## Authors’ contributions

Conception and design (MI, TO, KI, NK); data acquisition (TO, ME, YM), data analysis and interpretation (MI, TO, KY); writing and review of the manuscript (MI, KY, KI, NK). All authors read and approved the final manuscript.
